# UNDERSTANDING THE IMPACT OF COVID-19 ON THE USE OF TRANSPORTATION SERVICES BY OREGON ASSISTED LIVING RESIDENTS

**DOI:** 10.1093/geroni/igad104.2675

**Published:** 2023-12-21

**Authors:** Minju Kim, Ozcan Tunalilar, Paula Carder

**Affiliations:** Portland State University, Portland, Oregon, United States; Portland State University, Portland, Oregon, United States; Portland State University Institute on Aging, Portland, Oregon, United States

## Abstract

COVID-19 has affected many aspects of daily life, including transportation. This study aims to understand the impact of the COVID-19 pandemic on the use of transportation services by residents of assisted living communities in Oregon. Primary data about transportation service availability and use were collected from 721 AL communities using mailed questionnaires, before (winter of 2020) and during the pandemic (winter of 2022). To identify the impact of the pandemic on transportation use, we estimated a series of logistic regression models for trip purposes: medical appointments and social activities. Results show that the pandemic profoundly impacted transportation services, especially provided transportation services for access to social activities decreased from 77% to 68%, whereas access to medical appointments had not changed. The use of virtual visits allowed residents to remain connected with their loved ones while minimizing the risk of exposure to the virus. The findings of this study can help policymakers and transportation providers understand the impact of the pandemic on the mobility of individuals in community-based care settings. The results can also inform the development of strategies to improve transportation services and ensure the mobility of residents after the pandemic. Ultimately, the study can contribute to enhancing the overall quality of life for individuals in community-based care settings in Oregon.

